# Plasma FGF21 Concentration in Kidney Transplant Patients—Results from Prospective and Cross-Sectional Studies

**DOI:** 10.3390/jcm13144266

**Published:** 2024-07-22

**Authors:** Magdalena Bartmańska, Andrzej Wiecek, Marcin Adamczak

**Affiliations:** Department of Nephrology, Transplantation and Internal Medicine, Medical University of Silesia, 40-027 Katowice, Polandawiecek@sum.edu.pl (A.W.)

**Keywords:** FGF21, chronic kidney disease, kidney transplantation, metabolic disorders, fibroblast growth factor

## Abstract

**Background/Objectives:** Fibroblast growth factor 21 (FGF21) is a protein hormone involved in physiological conditions in the regulation of energy expenditure and several metabolic processes. The aim of this present study was to analyze the effect of successful kidney transplantations on the plasma FGF21 concentration and to study the factors which may influence plasma FGF21 concentration in patients in long time after kidney transplantation. **Methods:** This study consisted of two independent parts. The first part was a prospective observation of CKD patients in stage 5 before and then on the 14th and 30th day and 6 months after kidney transplantation. The second part of this study was the cross-sectional study completed in patients at least one year after kidney transplantation and the control group. In CKD patients directly before and during the early period after KTx, plasma FGF21 concentrations were measured four times (immediately before and 14 and 30 days and 6 months after KTx). In patients long time after kidney transplantation and in healthy subjects, plasma FGF21 concentration was measured once. **Results:** Forty patients with chronic kidney disease (CKD) who were either directly before or within the early period after kidney transplantation (KTx), 184 patients longtime after KTx and 50 healthy subjects were enrolled into this study. In CKD patients at the stage directly before receiving a KTx, the mean plasma FGF21 concentration was significantly higher than in the healthy subjects [1013.0 pg/mL versus 239.5 pg/mL, *p* < 0.001]. At 14, 30 days, and 6 months after the KTx, a significant decrease of plasma FGF21 was observed, with values of 322.5 pg/mL; 355.0 pg/mL; and 344.0 pg/mL (*p* < 0.001), respectively]. In patients long time after KTx, a negative correlation was found between the plasma FGF21 concentration and the estimated glomerular filtration rate and a positive correlation was found between the plasma FGF21 concentration and the BMI, the serum concentration of triglycerides, insulin, interleukin-6, CRP, and cystatin C. **Conclusions:** The plasma FGF21 concentration in patients with end-stage renal disease is higher than in healthy subjects and significantly decreases after a successful KTx. The plasma FGF21 concentration measured by ELISA in patients long time after kidney transplantation seems to be related to the degree of kidney function impairment and their metabolic status. The kidneys appear to be one of the main organs involved in the biodegradation and/or elimination of FGF21.

## 1. Introduction

The kidneys are involved in both the production, degradation and elimination of numerous hormones. Therefore, endocrine dysfunction might be a clinical problem in patients with chronic kidney disease (CKD) [1,2,3,4]. The abnormalities may concern the secretion, metabolism and biological activity of different hormones. The secretion of hormones by the kidneys (e.g., erythropoietin) and other endocrine organs (e.g., testosterone) is disturbed. In patients with CKD, due to reduced catabolism and/or renal clearance, some hormones are present in the plasma at increased concentrations (among others: gonadoliberin, LH, prolactin, PTH, insulin, leptin, adiponectin, resistin, and visfatin). Kidney disease may also disrupt the biological activity of hormones. The prohormone activation is disturbed and receptor resistance occurs. Therefore, symptoms of hormone deficiency might be observed in patients with CKD, despite the increased plasma concentration of numerous hormones. Most, but not all, hormonal imbalances in patients with CKD may improve after successful kidney transplantation.

Fibroblast growth factors are proteins that contain a core domain of 120 amino acids. Fibroblast growth factors act by binding to membrane fibroblast growth factor receptors (FGFRs). Despite their similar structure, fibroblast growth factors are proteins with different actions. Fibroblast growth factors (FGFs) take a role in embryonic development regulation, tissue regeneration processes after injuries, angiogenesis, and calcium-phosphate homeostasis, but also affect several metabolic processes [5]. Disturbances in the stimulation of fibroblast growth factor receptors may participate in the pathogenesis of neoplasms and metabolic diseases. Most fibroblast growth factors show a paracrine effect. However, proteins of the FGF19 subfamily, which include FGF19, FGF21, and FGF23, are secreted into the blood and exert endocrine effects [6].

FGF21 is a protein composed of 181 amino acids (~22.3 kDa) [6,7,8,9]. FGF21 is produced by the liver, adipose tissue, skeletal muscle, and the pancreas. The expression of the fgf21 gene is controlled by the peroxisome proliferator-activated receptors (PPARs). PPAR-α is found in the liver and PPAR-γ in adipose tissue cells. The liver is the main site of FGF21 production, while the adipocytes of white adipose tissue are its main site of action [7]. The FGF21 hepatic secretion is stimulated in situations of metabolic stress such as prolonged starvation [8]. The activity of FGF21 depends on its binding to the FGFR with β-Klotho as a cofactor. Expression of the β-Klotho protein is mainly detected in metabolically active organs (liver, white adipose tissue, and pancreas). Both the liver and white adipose tissue express the FGFR and β-Klotho, making these two organs the main site of FGF21 action [9,10].

Animal experiments have shown a beneficial effect of FGF21 on the metabolic profile, including increasing insulin sensitivity [11]. In animal studies, it was also found that the administration of FGF21 to mice with high-fat diet-induced obesity reduces their body weight by increasing energy expenditure and reducing glycemia, and leads to the reduction of hepatic steatosis [12]. FGF21 increases hepatic and peripheral insulin sensitivity in obese and non-obese mice [13]. FGF21 increases glucose uptake by the adipocytes in an insulin-independent manner. Unlike the immediate effect of insulin, this effect becomes apparent 4 h after the FGF21 administration [11,14]. In experimental animals under starvation or during ketogenic diets, an increased expression of FGF21 contributes to gluconeogenesis, ketogenesis, and hepatic fatty acid oxidation [15]. Although the FGF21 response to starvation is rapid in mice, the situation is quite different in humans. In humans, it was shown that FGF21 secretion increases after longer fasting for over a week (7 to 10 days). This is associated with a reduction in thermogenesis and adiponectin, and does not stimulate starvation-induced ketogenesis [16]. Studies suggest that since the liver is one of the main organs responsible for the secretion of FGF21, its concentration increases in conditions of liver congestion such as congestive heart failure [17].

On the other hand, FGF21 protects pancreatic β cells [18]. Administration of FGF21 improves the lipid profile by increasing the high-density lipoprotein serum concentration and by reducing the serum low-density lipoprotein concentration [19]. In white adipose tissue, FGF21 increases the expression of the thermogenin coactivator-1α PPARγ mRNA and the hormone dependent triglyceride lipase of adipocytes [15]. It has been shown that in transgenic mice with an overexpression of the fgf21 gene, a high-fat diet does not lead to the development of obesity [11]. In clinical studies, a higher plasma FGF21 concentration has been found in obese patients with metabolic disorders, impaired glucose tolerance, dyslipidemia, nonalcoholic liver disease, and type 2 diabetes [20,21,22]. In phase 2b trials, treatment of nonalcoholic steatohepatitis (NASH) with pegozafermin (a glycopegylated fibroblast growth factor 21 analog) led to improvements in fibrosis. It has been documented that among 219 patients receiving pegozafermin or a placebo, the fibrosis improvement was observed in 7% of those receiving a placebo, and 22% and 26% of those receiving the FGF21 analog in doses, respectively, of 15 mg and 30 mg [23].

Studies carried out so far have shown a significant relationship between the plasma FGF21 concentration and kidney function. Lin et al. demonstrated in a group of 240 subjects (200 patients with chronic kidney disease at various stages and 40 healthy subjects) that the plasma FGF21 concentration increases with the deterioration of kidney function [24]. Gonzalez et al. assessed the FGF21 plasma concentration in 48 CKD patients starting treatment with peritoneal dialysis. A positive correlation was shown between the plasma FGF21 concentration and the duration of dialysis treatment. It was also found that patients with preserved residual kidney function were characterized as having significantly lower plasma FGF21 concentrations compared to patients with anuria [25]. Moreover, Han et al. showed an eight times higher FGF21 plasma concentration in patients treated with peritoneal dialysis (*n* = 72) in comparison to healthy subjects (*n* = 63) [26]. Stein et al. demonstrated in their study, which included 60 patients with end-stage kidney disease treated with haemodialysis, that plasma FGF21 concentrations was 15 times higher than in 60 appropriately selected healthy subjects [27]. Hindricks et al. showed a significantly elevated plasma FGF21 concentration in patients after unilateral, partial, or complete nephrectomy [28]. A clinical study by Crasto et al. documented that the FGF21 plasma concentration is related to kidney function [29]. It has also been shown that an increased FGF21 plasma concentration in haemodialysis patients may be a prognostic factor for premature death, regardless of the concomitant risk of cardiovascular disease [30]. CKD patients, as mentioned earlier, have significantly increased plasma FGF21 concentrations.

The results of the abovementioned clinical studies showed that an increase in the FGF21 plasma concentration in CKD patients is due to a significant impairment of kidney excretory function, which leads to a decrease in the FGF21 clearance [25,27,28]. In addition, in patients with CKD a reduced level of the β-Klotho protein and the number of FGFR1c receptors was demonstrated, which may result in resistance to FGF21 [31].

In contrast to the CKD patients the issue of FGF21 was not adequately studied in kidney transplant patients. Only a few studies have investigated the FGF21 plasma concentration in small groups of patients after kidney transplantation [32].

The aim of this present clinical study was to analyze the effect of a successful kidney transplantation on the plasma FGF21 concentration measured by ELISA. Due to the fact that kidney transplant recipients are at the increased risk of metabolic disorders we wanted to analyze the factors related to plasma FGF21 concentration in patients long time after kidney transplantation.

## 2. Material and Methods

This study’s protocol was approved by the Bioethics Committee of the Medical University of Silesia in Katowice, Poland (KNW/0022/KB1/83/13). This study consisted of two independent parts.

1. The first part was a prospective observation of CKD patients in stage 5 before and then on the 14th and 30th day and 6 months after kidney transplantation. In this part of the study, patients with CKD stage 5 immediately before kidney transplantation were included. In all these patients blood samples were collected four times (directly before kidney transplantation, on the 14th and 30th day after kidney transplantation, and 6 months after kidney transplantation). The serum concentrations of FGF21 and creatinine were estimated.
2. The second part of this study was the cross-sectional study completed in patients at least one year after kidney transplantation and the control group.
  In this part of the study blood samples were collected once and the plasma concentrations of FGF21 and serum creatinine were measured. Serum concentrations of glucose, triglycerides, total cholesterol, insulin, cystatin C, CRP, interleukin 6, and the blood concentration of immunosuppressants were measured in patients long time after transplantation only. A metabolic syndrome (MS) was diagnosed in patients fulfilling *NCEP—ATP III* criteria (The National Cholesterol Education Program, Adult Treatment Panel III) i.e., any three of the following five criteria constitute a diagnosis of metabolic syndrome:
3. waist circumference: ≥102 cm in men and ≥88 in women
4. TG: ≥150 mg/dL (1.7 mmol/L) or drug treatment for elevated TG
5. HDL-C: <40 mg/dL (1.03 mmol/L) in men and <50 mg/dL (1.3 mmol/L) in women
6. blood pressure: ≥130 mm Hg systolic BP, or ≥85 mm Hg diastolic BP, or drug treatment for hypertension
7. fasting glucose: ≥100 mg/dL or pharmacological antidiabetic therapy

eGFR was measured using a MDRD formula and a HOMA index with the formula below:

(1) $$HOMA=\frac{fasting\;insulin\;concentration\left[\frac{\mu U}{mL}\right]\times fasting\;glucose\;concentration\left[\frac{mmol}{L}\right]}{22.5}$$

The FGF21 plasma concentration was measured using a Human FGF21 ELISA (BioVendor R&D, Brno, Czech Republic). The cystatin C serum concentration was measured using a Human Cystatin C ELISA (BioVendor R&D, Brno, Czech Republic). The other parameters were measured with the use of routine laboratory methods.

The FGF21 serum concentration was determined by the ELISA test only, which is characterized by high sensitivity.

The obtained results were subjected to a statistical analysis, calculating the median and confidence interval (95% CI). Most parameters were not normally distributed, therefore non-parametric tests were used in the statistical analysis. The degree of statistical variability of the difference between groups was determined using the Mann–Whitney U test and the Kruskal–Wallis test with a post-hoc analysis. The correlation coefficient was calculated according to Spearman. A comparison of changes in the concentrations of the determined parameters in patients immediately after kidney transplantation in the prospective follow-up was performed using Friedman’s ANOVA and Dunn’s post-hoc analysis. The differences between the examined values were considered statistically significant with a *p* value < 0.05. Statistical analysis was performed using the Statistica 10.0 PL program for Windows (StatSoft Polska, Kraków, Poland).

## 3. Results

In the first part of this study, 40 patients with CKD 5 were enrolled (26 women and 14 men). The characteristics of this group are shown in Table 1. All 40 patients from this part of the study received a kidney from a deceased donor. The median donor’s age was 47.5 years (41.0–50.0 95% CI). All patients received as immunosuppressive therapy: tacrolimus, steroids, and mycophenolate mofetil. In 31 patients (77.5%) a graft biopsy was carried out between 7 and10 days after transplantation. In five of them features of acute rejection were found (two acute vascular rejections, two acute cellular tubulointerstitial rejections, and one acute humoral rejection).

In the second part of this study, 184 patients were involved (72 women and 112 men), and the characteristics of this group are presented in Table 2.

The control group consisted of 50 volunteers with normal kidney function, and the characteristics of the control group are shown in Table 2.

**Figure 1:**
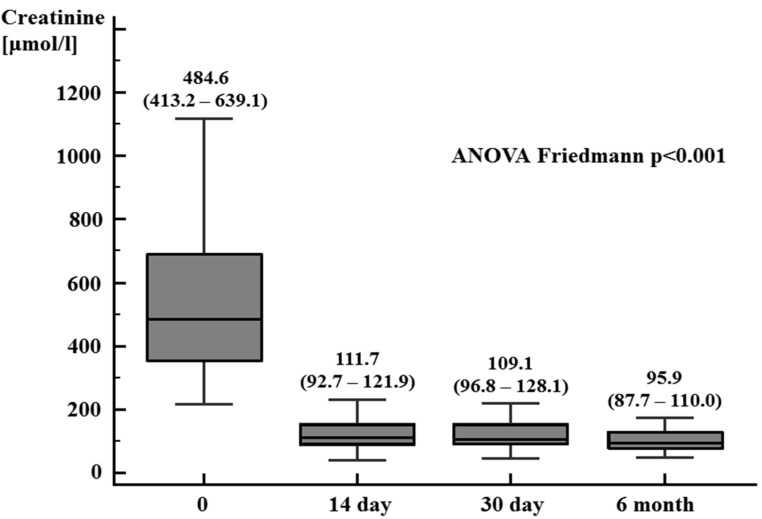
Serum creatinine concentration in patients after kidney transplantation (0, 14, and 30 days and 6 months after the procedure) (median, 95% CI)—first part of the study.

**Figure 2:**
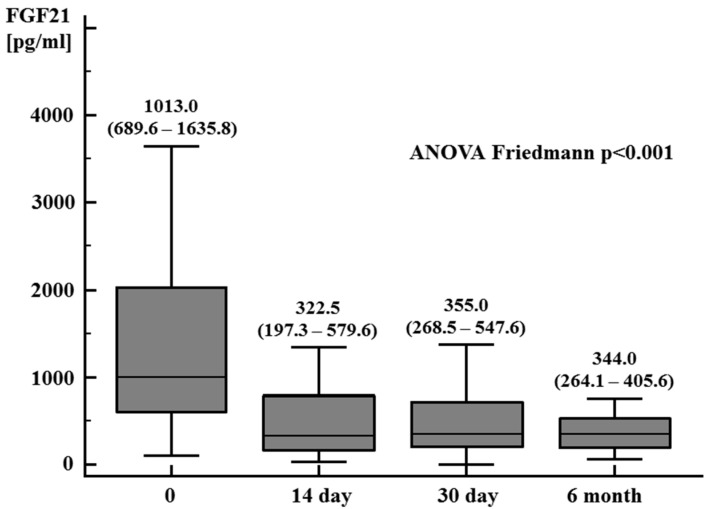
FGF21 plasma concentration in patients after kidney transplantation (0, 14, and 30 days and 6 months after the procedure) (median, 95% CI)—first part of the study.

In the first part of this study, in kidney transplant patients, a significant reduction in serum creatinine was observed on the 14th and 30th day and 6 months after the transplantation in comparison to the results obtained before the kidney transplantation (Figure 1). The eGFR on the 14th and 30th day after the procedure was 48.5 mL/min/1.73 m^2^ (38.7–56.9 mL/min/1.73 m^2^) and 49.4 mL/min/1.73 m^2^ (43.0–56.1 mL/min/1.73 m^2^), respectively, and half a year after kidney transplantation was 53.5 mL/min/1.73 m^2^ (50.2–63.1 mL/min/1.73 m^2^) (ANOVA Friedmann *p* < 0.01). In patients with end-stage kidney disease, before kidney transplantation, a significantly higher plasma FGF21 concentration was observed compared to the control group, with values of 1013.0 pg/mL (689.6–1635.8 pg/mL) versus 239.5 pg/mL (219.0–294.5 pg/mL), respectively, *p* < 0.0001. A significant reduction in the plasma FGF21 concentration was demonstrated on the 14th and 30th days and 6 months after kidney transplantation (Figure 2).

Patients long time after kidney transplantation and healthy controls did not differ significantly in their cholesterol and glucose serum concentrations, or in their FGF21, insulin, and C-reactive protein plasma concentrations (Table 3). In patients long time after kidney transplantation, serum creatinine and cystatin C levels were significantly higher and the eGFR was significantly lower compared to the control group. In addition, patients after kidney transplantation were characterized by significantly elevated serum triglycerides and interleukin 6 concentrations compared to the control group (Table 3).

There were no correlations between the FGF21 serum concentration and any of the immunosuppressive drugs. Patients treated with cyclosporin A were significantly older compared to patients treated with tacrolimus (Table 4). Patients in both groups did not differ in terms of metabolic indicators (i.e., serum concentrations of triglycerides, cholesterol, glucose and insulin, as well as waist circumference, and HOMA index) or inflammatory parameters (interleukin 6 and CRP plasma concentration). The plasma FGF21 concentration was lower in patients receiving tacrolimus than in patients treated with cyclosporin A (Table 4). In patients treated with tacrolimus, serum creatinine and cystatin C concentrations were lower and the eGFR was higher compared to patients treated with cyclosporin A (Table 4). Therefore, an additional case-control analysis was carried out. Patients using cyclosporin A (*n* = 48) and tacrolimus (*n* = 48) were matched in terms of their eGFR. In such an analysis there was no significant difference in the FGF21 plasma concentration between the group treated with cyclosporin A and those treated with tacrolimus (median, 95% CI: 345 pg/mL [279.5–462.2 pg/mL] and 314.0 pg/mL [262.0–480.1 pg/mL], *p* = 0.657, respectively).

A metabolic syndrome was diagnosed in 76 (41.3%) patients long time after kidney transplantation group. Patients with and without a metabolic syndrome differ in age, eGFR, serum creatinine and cystatin C concentrations, and plasma interleukin 6 concentration (Table 5). There was also no significant difference in the FGF21 plasma concentration in patients with and without a metabolic syndrome (Table 5). Additionally, a case-control analysis matching patients with MS and without MS in terms of eGFR was carried out. Also in this analysis, no statistically significant difference was found in the plasma FGF21 concentration in patients with or without MS, with values of 316 pg/mL (272.6–406.2) and 247.5 pg/mL (188.0–324.0 pg/mL) (*p* = 0.053), respectively.

There was a significant difference in the plasma FGF21 concentration in patients long time after kidney transplantation, who were divided into two groups by their median BMI i.e., 25.9 kg/m^2^ (Table 6). Among the patients with a BMI above the median of BMI, the FGF21 plasma concentration was higher compared to the patients with a BMI under the median (*p* < 0.05). These patients did not differ significantly in their eGFR and serum cystatin C concentrations (Table 6).

Diabetes was diagnosed in 42 (23%) patients long time after kidney transplantation. Diabetic patients had significantly higher FGF21 and insulin plasma concentrations, higher fasting serum glucose concentrations and HOMA index, and greater waist circumferences compared to non-diabetic patients (Table 7). Diabetic patients were older than non-diabetic patients. The kidney graft function, assessed with the eGFR, creatinine and cystatin C serum concentrations was similar in patients in both groups (Table 7).

In patients long time after kidney transplantation, positive correlations were observed between the plasma FGF21 concentration and the following: BMI (R = 0.160; *p* < 0.05); serum triglyceride concentration (R = 0.396; *p* < 0.0001); plasma insulin concentration (R = 0.168; *p* < 0.05); interleukin 6 plasma concentration (R = 0.206; *p* < 0.005); plasma C-reactive protein concentration (R = 0.177; *p* < 0.05); and serum cystatin C concentration (R = 0.259; *p* < 0.001). A significant negative correlation was found between the plasma FGF21 and eGFR (R = −0.169; *p* < 0.05). Due to the fact that long after kidney transplantation diabetic patients were treated with insulin, a separate correlation analysis was also performed in non-diabetic patients. In the group of patients after kidney transplantation without diabetes, a positive correlation was found between the plasma concentration of FGF21 and the serum concentration of triglycerides (R = 0.422; *p* < 0.001), plasma insulin (R = 0.172; *p* < 0.05), plasma concentration of interleukin 6 (R = 0.171; *p* < 0.05), and serum cystatin C concentration (R = 0.254; *p* < 0.005).

## 4. Discussion

In the current study, a decrease in FGF21 plasma concentration was found in CKD patients who underwent successful kidney transplantation in comparison to the FGF21 plasma concentration measured immediately before kidney transplantation. In patients long time after kidney transplantation, significant relationships between the FGF21 plasma concentration and the transplanted kidney function, as well as, serum concentrations of insulin, interleukin 6, triglycerides and cystatin C, and plasma concentrations of C-reactive protein and BMI were demonstrated.

In patients with impaired kidney function, numerous disorders of endocrine organs resulting in abnormal synthesis, secretion, and metabolism of hormones are observed. These disorders tend to worsen as the CKD progresses. After successful kidney transplantation the severity of most hormonal abnormalities decreases [1,2]. In this study, a significant reduction in the plasma FGF21 concentration, along with an improved kidney graft function, was found in patients after successful kidney transplantations. The FGF21 plasma concentration decreases within 14 days after surgery. These results cannot be compared with any studies to date, since changes in FGF21 plasma concentration have not yet been evaluated in patients in the early post-transplant period.

The results of a few clinical studies suggest that the plasma FGF21 concentration depends on kidney function. Higher concentrations of plasma FGF21 were observed in patients with CKD compared to healthy populations [24,25,26,27,28]. Stein et al. documented that as CKD progresses, the plasma FGF21 concentration increases, reaching the highest values in patients treated with haemodialysis [27]. In patients treated with peritoneal dialysis, the plasma FGF21 concentration, although higher than in the healthy population, is lower than in patients undergoing haemodialysis. One may speculate that FGF21 passes through the peritoneal membrane. It may reduce the plasma FGF21 concentration. Lower plasma concentrations of FGF21 in patients treated with peritoneal dialysis may be also associated with better residual renal function. Gonzalez et al. showed that patients treated with peritoneal dialysis with residual kidney function (RRF) were characterized by lower FGF21 plasma concentrations compared to patients without RRF [25]. In this current study, we also found a higher plasma FGF21 concentration in patients with end-stage kidney disease (directly before kidney transplantation) compared to healthy subjects. Therefore, it is likely that in patients with an impaired kidney function, FGF21, like other protein hormones, accumulates in the plasma due to its decreased kidney clearance. In addition, an increased plasma FGF21 concentration in patients treated with dialysis may also be associated with concomitant chronic inflammation, insulin resistance, or obesity [30,33,34].

The results of this current study suggest that the plasma FGF21 concentration in the long-term follow-up after kidney transplantation depends on the kidney function and metabolic status.

The clinical studies conducted so far, have shown that the plasma FGF21 concentration depends on kidney function [24,25,26,27,28]. Also in the present study, a negative correlation was found between the eGFR and FGF21 plasma concentration in patients long time after kidney transplantation. Cystatin C is freely filtered through the glomeruli, then almost completely reabsorbed, and catabolized by the tubular epithelial cells (>99%). Therefore, its plasma concentration correlates with glomerular filtration [35]. This current study showed a positive correlation between the serum cystatin C concentration and plasma FGF21 concentration in patients long time after kidney transplantation.

It is thought that FGF21 appears to play a role in the response to metabolic stress by creating adaptive mechanisms. Factors causing so-called metabolic stress are, among others, obesity, hyperglycemia, and chronic inflammation [33]. The clinical trials conducted so far have shown an increased risk of developing metabolic disorders in patients after organ transplantation, including kidney transplantation [36,37,38,39,40]. In the present study, a higher serum triglyceride concentration was found in patients long time after after kidney transplantation compared to healthy subjects. In this current study there were no differences between the FGF21 plasma concentrations in patients long time after kidney transplantation with or without MS. Due to the difference in glomerular filtration between patients with MS and without MS, the case-control analysis was additionally completed (patients were matched with eGFR). Also, in this analysis there were no differences in the FGF21 plasma concentration in patients with and without a metabolic syndrome.

To date, only a few studies have been conducted to assess the plasma FGF21 concentration in patients long time after kidney transplantation. Bagheri et al. [41] studied the relationship between the plasma FGF21 concentration and the occurrence of a metabolic syndrome in patients after kidney transplantation. This study consisted of 86 patients with a stable graft function at least 6 months after kidney transplantation. There was no significant difference in the plasma FGF21 concentrations in patients after kidney transplantation with MS compared to patients without MS. Therefore the results of the abovecited study are consistent with the results obtained in our current study.

Disorders of carbohydrate metabolism are frequently found in patients with end-stage kidney disease and after kidney transplantation. These patients often develop insulin resistance and then diabetes [38,39,42]. In animal experiments and in clinical studies, it was found that the plasma FGF21 concentration is higher in obese subjects, as well as those with a metabolic syndrome and type 2 diabetes. It might occur due to the FGF21 receptors resistance in patients with impaired kidney function [22]. Chen et al. in a 5-year observation of the general population, showed that increased FGF21 plasma concentrations along with waist circumference and fasting glycaemia are an independent predictor of type 2 diabetes [43]. Woo et al. in a prospective study of 1380 subjects without diabetes from the general population, found that an increased plasma FGF21 concentration is the superior biomarker, among others such as adiponectin and A-FABP (adipocyte fatty acid-binding protein) plasma concentrations, based on which diabetes can be predicted [44].

In the present study, 42 patients (23%) long time after kidney transplantation, were diagnosed with diabetes mellitus. Diabetic patients were older than non-diabetic ones, but they did not differ in terms of their transplanted kidney function. It has been shown that patients with diabetes were characterized by a higher FGF21 plasma concentration compared to patients without diabetes. They also had a higher CRP plasma concentration and a higher waist circumference compared to patients without diabetes.

Our study showed a relationship between the plasma FGF21 and interleukin 6 concentrations. Interleukin 6 is a cytokine with pro-inflammatory properties [45]. It is suggested that FGF21 may play a role in the inflammatory processes. Gariani et al. showed an increased plasma FGF21 concentration in patients with sepsis and systemic inflammatory response syndrome. Along with a clinical improvement, the plasma FGF21 concentration decreased in these patients [46]. Moreover, results of recent studies suggest an interference of FGF21 with anti-inflammatory and anti-oxidation processes [47,48]. In this current study, both the FGF21 and interleukin 6 plasma concentrations were increased in patients long time after kidney transplantation with MS. Therefore it might be associated with metabolic disorders and low-grade chronic inflammation. In the present study, an increased plasma concentration of interleukin 6 in the patients long time after kidney transplantation was observed compared to the control group. It could be explained by co-existing metabolic disorders and chronic inflammation.

The immunosuppressive drugs used after kidney transplantation significantly disturbed carbohydrate metabolism. Tacrolimus may participate in the development of new-onset diabetes mellitus after transplantation. In animal and clinical studies, tacrolimus has been shown to reduce insulin secretion [49]. Tacrolimus has a stronger diabetogenic effect compared to cyclosporin A [50,51]. In our study, when patients were matched by the eGFR, there was a difference in plasma FGF21 concentrations between patients from both groups. Founded in the results section, in the initial analysis a higher plasma FGF21 concentration in patients receiving cyclosporin A was only caused by a significantly worse kidney transplant function.

The limitation of this study, especially the first part, was that the number of patients was too low, which did not allow for analyses in subgroups or correlational analyses. The limitation of the second part of this study is the fact that it included only a cross-sectional analysis.

Based on the results of this current study it may be concluded that the plasma FGF21 concentration in patients with end-stage renal disease is higher than in healthy subjects and significantly decreases after successful kidney transplantation. The plasma FGF21 concentration in patients long time after kidney transplantation is related to the degree of impairment to renal function and their metabolic status. The kidneys appear to be one of the main organs involved in the biodegradation and/or elimination of FGF21.

## Figures and Tables

**Table 1:** Characteristics of patients before kidney transplantation (first part of the study) (median 95% CI, percentage for categorizing variables).

Number of Patients	40
Females	26 (65%)
Males	14 (35%)
Age [years]	47.0 (39.2–54.0)
Patients on haemodialysis	38 (95%)
Patients on peritoneal dialysis	1 (2.5%)
Time of dialysis [month]	24 (21.0–30.1)
Patients before initiation of RRT	1 (2.5%)
BMI [kg/m^2^]	24.8 (22.0–25.9)
Cause of CKD	Chronic glomerulonephritis *n* = 16 (40%) Hypertensive nephropathy *n* = 6 (15%) Diabetic kidney disease *n* = 4 (10%) Vasculitis *n* = 4 (10%) Chronic tubulointerstitial nephritis *n* = 3 (7.5%) Polycystic kidney disease *n* = 2 (5%) Alport syndrome *n* = 1 (2.5%) Familial hypomagnesemia with Hypercalciuria and Nephrocalcinosis *n* = 1 (2.5%) Undetermined *n* = 2 (5%)

RRT—renal replacement therapy. BMI—body mass index.

**Table 2:** Characteristics of patients long time after kidney transplantation and the control group (second part of the study) (median 95% CI, percentage for categorizing variables).

	Patients Long Time after Kidney Transplantation	Control Group
Number of patients	184	50
Females	72 (39.1%)	28 28 (56%)
Males	112 (60.9%)	22 22 (44%)
Age [years]	52.0 (48.0–54.0)	52.0 (48.0–58.0)
BMI [kg/m^2^]	25.9 (25.1–26.7)	26.7 (25.9–27.1)
Waist circumference [cm]	93.0 (91.2–96.0)	91.0 (85.0–95.4)
Time after kidney transplantation [month]	49.0 (40.2–59.7)	
Immunosuppressive therapy with drug blood concentration [ng/mL]	Tacrolimus *n* = 135 (73%) 6.5 (6.2–6.9) Cyclosporin A *n* = 49 (27%) 89.5 (78.7–101.28)	
Cause of CKD	Chronic glomerulonephritis *n* = 65 (35.3%) Hypertensive nephropathy *n* = 14 (7.6%) Polycystic kidney disease *n* = 12 (6.5%) Pyelonephritis *n* = 7 (3.8%) Diabetic kidney disease *n* = 4 (2.2%) Nephrolithiasis *n* = 2 (1.1%) Others * *n* = 16 (8.7%) Undetermined *n* = 64 (34.8%)	

BMI—body mass index, * cancer, systemic vasculitis, nephrocalcinosis, congenital malformations.

**Table 3:** Creatinine, glucose, cholesterol, triglycerides, and cystatin C serum concentrations; and FGF21, insulin, C-reactive protein, and interleukin 6 plasma concentrations; and eGFR, HOMA, waist circumference, BMI, and age in patients long time after kidney transplantation and in the control group (median, 95% CI).

	Patients in the Long Time after Kidney Transplantation	Control Group	*p*
Number	184	50	
Age [year]	52.0 (48.0–54.0)	52.2 (48.0–58.0)	0.332
Creatinine [µmol/L]	114.0 (106.7–124.0)	71.8 (70.0–76.7)	<0.0001
eGFR [mL/min/1.73m^2^]	51.2 (48.2–57.9)	77.9 (76.1–85.2)	<0.0001
Cystatin C [ng/mL]	1778.5 (1662.4–1874.0)	896.0 (754.4–1103.0)	<0.0001
Glucose [mmol/L]	5.2 (5.0–5.4)	5.0 (4.9–5.2)	0.135
Cholesterol [mmol/L]	5.2 (5.0–5.4)	5.4 (4.9–5.6)	0.976
Triglycerides [mmol/L]	1.6 (1.4–1.7)	1.3 (1.1–1.4)	<0.05
FGF21 [pg/mL]	299.0 (268.2–343.6)	239.5 (219.0–294.5)	0.256
CRP [mg/L]	3.1 (2.4–3.9)	3.7 (2.6–4.6)	0.444
Interleukin 6 [pg/mL]	2.7 (2.3–3.0)	1.4 (1.2–1.7)	<0.0001
Insulin [µU/mL]	9.4 (8.5–10.0)	9.2 (8.2–9.8)	0.232
BMI [kg/m^2^]	25.9 (25.1–26.7)	26.7 (25.9–27.1)	0.445
HOMA	2.2 (2.0–2.5)	2.0 (1.9–2.3)	0.123
Waist circumference [cm]	93.0 (91.2–96.0)	91.0 (85.0–95.4)	0.061

eGFR—estimated glomerular filtration, FGF21—fibroblast growth factor 21, CRP—C-reactive protein, HOMA—homeostatic model assessment, BMI—body mass index.

**Table 4:** Creatinine, cystatin C serum concentration, FGF21 plasma concentration, eGFR, and age in patients long time after kidney transplantation treated with tacrolimus or cyclosporin A (median, 95% CI).

	Patients Treated with Tacrolimus	Patients Treated with Cyclosporin A	*p*
Number	135	49	
Age [year]	48.0 (45.0–52.0)	56.5 (54.0–59.3)	<0.01
Creatinine [µmol/L]	107.0 (103.4–120.8)	136.7 (116.4–149.6)	<0.02
eGFR [ml/min/1.73 m^2^]	53.4 (48.6–60.4)	46.4 (37.0–54.4)	<0.02
Cystatin C [ng/mL]	1614.0 (1538.7–1703.3)	2030.0 (1892.6–2367.2)	<0.001
FGF21 [pg/mL]	288.0 (247.6–333.5)	348.0 (280.5–480.5)	<0.05

eGFR—estimated glomerular filtration, FGF21—fibroblast growth factor 21.

**Table 5:** Creatinine, glucose, cholesterol, triglycerides, and cystatin C serum concentrations; and FGF21, insulin, C-reactive protein, and interleukin 6 plasma concentrations; eGFR, HOMA, waist circumference, BMI, and age in patients long time after kidney transplantation with a metabolic syndrome and without this syndrome (median, 95% CI).

	Patients without Metabolic Syndrome	Patients with Metabolic Syndrome	*p*
Number	108	76	
Age [year]	46.0 (42.0–50.6)	55.0 (52.0–58.0)	<0.005
Creatinine [µmol/L]	106.9 (102.0–117.5)	129.8 (115.2–143.1)	<0.05
eGFR [mL/min/1.73m^2^]	54.3 (49.0–63.6)	47.9 (43.5–53.5)	<0.05
Cystatin C [ng/mL]	1666.0 (1570.3–1866.3)	1840.5 (1715.7–1983.6)	0.139
Glucose [mmol/L]	4.9 (4.8–5.0)	6.1 (5.9–6.4)	<0.001
Cholesterol [mmol/L]	5.1 (4.9–5.5)	5.3 (5.0–5.5)	0.441
Triglycerides [mmol/L]	1.4 (1.3–1.5)	1.8 (1.7–2.0)	<0.001
FGF21 [pg/mL]	278.0 (242.3–337.9)	326.5 (278.0–404.1)	0.119
CRP [mg/L]	2.8 (1.9–3.6)	4.0 (2.5–5.6)	0.061
Interleukin 6 [pg/mL]	2.5 (2.2–2.8)	3.3 (2.3–3.9)	<0.05
Insulin [µU/mL]	8.0 (7.2–8.7)	11.7 (10.8–13.1)	<0.001
BMI [kg/m^2^]	24.4 (23.9–25.5)	27.7 (26.7–29.2)	<0.001
HOMA	1.7 (1.6–1.9)	3.2 (2.8–4.0)	<0.001
Waist circumference [cm]	88.5 (87.0–92.0)	100.0 (928.0–106.0)	<0.001

eGFR—estimated glomerular filtration, FGF21—fibroblast growth factor 21, CRP—C-reactive protein, HOMA—homeostatic model assessment, BMI—body mass index.

**Table 6:** Creatinine, glucose, cholesterol, triglycerides, cystatin C serum concentrations; and FGF21, insulin, C-reactive protein and interleukin 6 plasma concentrations, eGFR, HOMA, waist circumference, BMI and age in patients long time after kidney transplantation, median—divided BMI (median, 95% CI).

	Patients with BMI < Median	Patients with BMI > Median	*p*
Number	92	92	
Age [year]	47.0 (42.0–52.0)	55.0 (52.2–57.0)	<0.05
Creatinine [µmol/L]	109.3 (103.6–122.8)	121.0 (106.7–130.8)	0.631
eGFR [mL/min/1.73 m^2^]	56.3 (49.7–63.8)	48.2 (45.8–53.4)	0.229
Cystatin C [ng/mL]	1714.0 (1607.7–1891.8)	1806.0 (1659.7–1936.8)	0.415
Glucose [mmol/L]	4.9 (4.8–5.1)	5.6 (5.3–6.0)	<0.001
Cholesterol [mmol/L]	5.0 (4.9–5.4)	5.2 (5.1–5.6)	0.504
Triglycerides [mmol/L]	1.4 (1.3–1.6)	1.6 (1.5–1.7)	<0.05
FGF21 [pg/mL]	257.0 (214.7–331.0)	330.0 (288.5–381.5)	<0.05
CRP [mg/L]	2.2 (1.6–3.3)	4.4 (3.0–5.3)	<0.005
Interleukin 6 [pg/mL]	2.6 (2.1–3.0)	2.8 (2.3–3.7)	0.210
Insulin [µU/mL]	7.4 (6.6–8.2)	11.4 (10.4–13.0)	<0.001
BMI [kg/m^2^]	23.6 (23.1–24.0)	29.0 (27.8–29.7)	<0.001
HOMA	1.6 (1.5–1.8)	2.9 (2.6–3.4)	<0.001
Waist circumference [cm]	87.0 (83.1–89.0)	101.0 (99.0–104.9)	<0.001

eGFR—estimated glomerular filtration, FGF21—fibroblast growth factor 21, CRP—C-reactive protein, HOMA—homeostatic model assessment, BMI—body mass index.

**Table 7:** Creatinine, glucose, cholesterol, triglycerides, cystatin C serum concentrations and FGF21, insulin, C-reactive protein and interleukin 6 plasma concentration, eGFR, HOMA, waist circumference, BMI, and age in patients long time after kidney transplantation with and without diabetes mellitus (median, 95% CI).

	Patients without Diabetes Mellitus	Patients with Diabetes Mellitus	*p*
Number	142	42	
Age [year]	50.0 (45.0–54.0)	56.5 (50.0–58.0)	<0.05
Creatinine [µmol/L]	114.0 (106.2–125.0)	114.4 (98.1–135.7)	0.790
eGFR [mL/min/1.73 m^2^]	51.2 (47.7–58.8)	51.9 (42.2–66.1)	0.978
Cystatin C [ng/mL]	1711.0 (1614.0–1868.4)	1865.5 (1689.7–2085.6)	0.208
Glucose [mmol/L]	5.0 (4.9–5.2)	6.9 (5.8–7.7)	<0.001
Cholesterol [mmol/L]	5.2 (5.0–5.5)	5.0 (4.7–5.5)	0.441
Triglycerides [mmol/L]	1.6 (1.4–1.7)	1.6 (1.4–1.7)	0.805
FGF21 [pg/mL]	277.0 (246.4–324.0)	358.5 (300.7–515.0)	<0.05
CRP [mg/L]	2.8 (2.0–3.5)	4.8 (3.0–7.2)	<0.05
Interleukin 6 [pg/mL]	2.7 (2.2–4.7)	2.3 (2.0–4.0)	0.181
Insulin [µU/mL]	9.3 (8.4–10.5)	10.6 (7.5–13.1)	0.406
BMI [kg/m^2^]	25.3 (24.8–26.1)	27.9 (26.0–29.7)	<0.01
HOMA	2.1 (1.9–2.3)	3.0 (2.2–4.8)	<0.05
Waist circumference [cm]	93.0 (90.0–95.0)	98.5 (92.1–106.0)	<0.01

eGFR—estimated glomerular filtration, FGF21—fibroblast growth factor 21, CRP—C-reactive protein, HOMA—homeostatic model assessment, BMI—body mass index.

## Data Availability

The data are not publicly available due to not covered by the Bioethics Committee’s approval for the study.
